# Is work burden associated with postmenopausal breast cancer? A population-based 25-year follow-up

**DOI:** 10.1007/s00404-024-07867-7

**Published:** 2024-12-12

**Authors:** Maria Kalliosaari, T. Rikkonen, R. Sund, M. Tuppurainen

**Affiliations:** 1 Department of Obstetrics and Gynecology, Wellbeing Services County of Central Finland/Hospital Nova of Central Finland, Hoitajantie 3, 40620 Jyvaskyla, Finland; 2 Kuopio Musculoskeletal Research Unit, University of Eastern Finland, Kuopio, Finland; 3 Department of Obstetrics and Gynecology, Kuopio University Hospital, Kuopio, Finland

**Keywords:** Breast cancer, Work burden, Sedentary work, MHT, Family history, Alcohol

## Abstract

**Objective:**

To study the association between breast cancer and work burden over 25 years.

**Methods:**

The study was based on the Kuopio Osteoporosis Risk Factor and Prevention (OSTPRE) cohort (n = 14,220) and included women who had answered the questionnaire from the year 1994 and had no previous breast cancer. Breast cancer cases were recorded from the Finnish Cancer Registry during the study period: from 1st June 1994 till December 31, 2019. Using questionnaires, we collected information on work burden, body mass index (BMI), menopausal hormone therapy (MHT), alcohol consumption, parity, and family history of breast cancer. Work burden was categorized as low or high. Variables were used both in the univariate and multivariate Cox regression analyses to explore their associations with breast cancer.

**Results:**

Altogether 825 women (6.9%) were diagnosed with breast cancer during the study period with a mean follow-up of 13.3 ± 7.2 years. Women with breast cancer were compared to those without breast cancer during the follow-up period (n = 11,117). A low work burden was associated with a 1.3-fold higher incidence of breast cancer (95% confidence interval 1.2–1.6) than a high work burden. Low work burden was associated with an increased breast cancer risk.

**Conclusion:**

Low work burden is associated with elevated postmenopausal breast cancer risk in the 25-year follow-up period.

## What does this study add to the clinical work

**Table:** 

Low self-rated work burden is related to the elevated risk of breast cancer in a 25-year follow-up study. Simultaneously, the strongest association with breast cancer was a positive family history of breast cancer and menopausal hormone therapy for 5–10 years in a population-based cohort of Finnish postmenopausal women	

## Introduction

Breast cancer (BC) is the most common cancer among women occurring worldwide [1] and the global cancer burden is increasing [2]. Simultaneously, mortality rates attributable to BC have decreased by nearly one-third in the past quarter century, the survival rate for BC has reached over 90% [1]. As the survival rate of BC is high, cancer affects the lives of hundreds of thousands of women worldwide. Possible modifiable risk and protective factors should be studied carefully to influence the growing incidence of BC.

There are several known risk factors for postmenopausal BC, including parity, family history, obesity, and nutritional factors such as alcohol consumption [3–7]. Recently, the possibility has been investigated that sedentary work might increase the risk of BC [8] or has no effect on it [9]. In contrast, nighttime work appears to increase the risk of BC [10, 11]. Because the findings of work burden and BC risk are conflicting and retirement age is rising worldwide while careers are longer [12, 13], it is important to further study the work burden regarding postmenopausal BC risk in a long-term follow-up.

The incidence of postmenopausal BC increases with age [1]. Because the role of work and the working environment in one’s life is growing, we wanted to investigate whether the role of work burden alters postmenopausal BC risk. The present study aimed to evaluate the role of self-rated work burden and other known risk factors for postmenopausal BC in a population-based 25-year prospective study among Finnish postmenopausal women.

## Materials and Methods

This cohort study was based on the Kuopio Osteoporosis Risk Factors and Prevention (OSTPRE) cohort. The OSTPRE is a population-based ongoing follow-up study, consisting of 14 220 women born in 1932–41, aged 47–56 years in the year 1989, living in Kuopio Province, Eastern Finland. The first questionnaire was mailed to these women in spring 1989. Follow-up questionnaires were sent at 5-year intervals (in 1994, 1999, 2004, 2009, 2014, and 2019) to women who responded to the baseline questionnaire. The response rates varied between 93% (baseline) and 75% (25 years).

The BC data were obtained from the Finnish Cancer Registry (FCR). Under Finnish legislation, all cancer cases are reported to the FCR by hospitals, physicians, and pathology laboratories. Also, the death certificates with the mention of cancer are combined with the FCR. The coverage of the FCR is almost 100% in solid tumors [14].

**Fig. 1:**
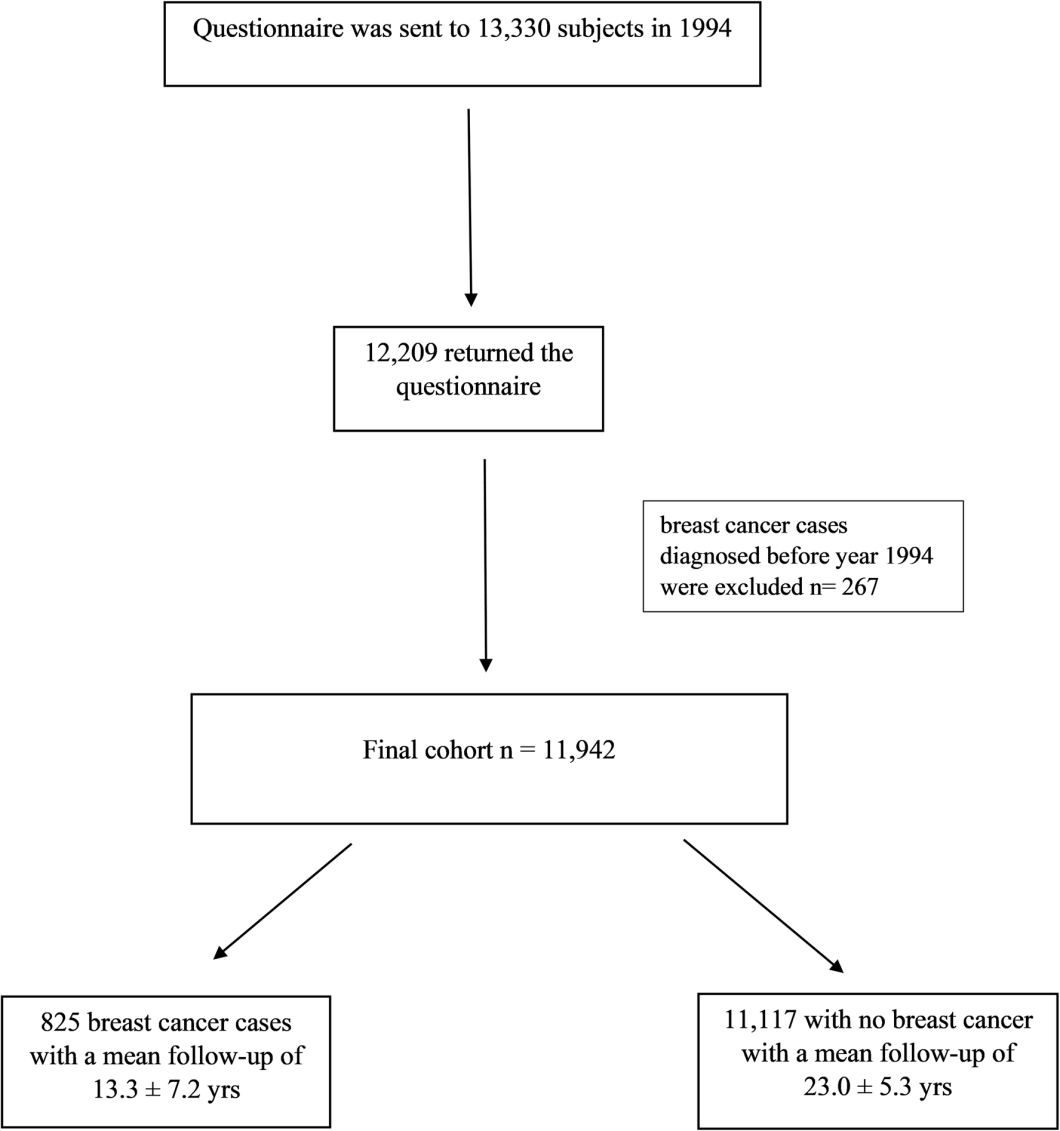
Flow chart of final cohort

In 1994, the 5th year OSTPRE follow-up questionnaire was sent to 13,330 women, of whom 12,209 responded. Patients with past history of breast cancer were excluded (n = 267) from the study population. Among the responders, 825 BC cases in 11,942 women were registered during the follow-up period from June 1, 1994, to December 31, 2019. Follow-up was terminated at the first occurrence of BC, emigration, death, or the end of follow-up (31st December 2019), whichever occurred first (Fig. 1).

### Questionnaires and variables

In this study, the 5-year postal inquiry from 1994 was regarded as the baseline because at this time, the work burden and family history (FH) of the BC history of the subject’s mother were inquired.

Work burden, occupation, and work history were recorded through self-reports. Initially, there were four (sedentary, low, intermediate, and high) options. Groups were recategorized by combining sedentary work and low work burden as “low work burden” while the “high work burden” group included intermediate and hard work. The low work burden category consisted of office workers, teachers, and secretaries. The high work burden category included occupations such as farmers, childminders/nursemaids, cleaners, housewives, and bakers. No information on shift work or possible night shifts was available.

The baseline health questionnaire also included additional information such as current weight (kg) and monthly alcohol consumption. Alcohol intake consisting of beer, wine, and spirits was converted into absolute ethanol consumption (g/week), with one dose equaling 12 g of absolute alcohol. Alcohol consumption (AC) groups were created according to moderate- to high-risk alcohol use in Finland [15].

The amount of smoking (cigarettes per day) and smoking history (smoking years) were converted to pack-years (years of smoking 20 cigarettes per day).

Combining variables from the 1989 inquiry, pregnancy, parity, and duration of past and present menopausal hormone therapy (MHT) use were registered and considered in the duration of MHT. Body mass index (BMI; kg/m^2^) was calculated using the current weights from the 1994 questionnaire. The date of the last menstruation was used to calculate the age at menopause for women who had intact ovaries and uteri. If the age at menopause was unknown because of removal of the ovaries or uterus, it was replaced with the mean menopausal age of the entire cohort.

### Statistical analysis

The statistical analyses were performed with the Statistical Package for Social Sciences program (IBM Corp. Released 2020. IBM SPSS Statistics for Windows, Version 27.0. Armonk, NY: IBM Corp). The chi-square test and one-way ANOVA were used to compare the differences between groups. The risk ratios in terms of hazard ratios (HR) were calculated with 95% confidence intervals (CIs) with uni- and multivariable Cox regression. Continuous variables are presented as means with standard deviations.

Self-rated work burden was used as a categorical variable, with low and high work burdens. The association between work burden and BC was adjusted for covariates, including age, BMI, FH of BC, and duration of MHT and AC at baseline in the multivariable analysis. Age and BMI were used as continuous variables in the model. Parity (none vs. any), FH (no vs. yes), duration of MHT (none, under 5 years, 5–10 years and over 10 years), and AC (none, 0.1–100 g, over 100 g/week) were treated as categorical variables. *P* values < 0.05 were considered statistically significant.

## Results

The study population consisted of 11,942 women aged 57.3 (± 2.9) years at baseline. The average age at menopause was 49.8 (± 4.3) years for women with intact uterus and ovaries. The mean follow-up for the whole cohort was 22.3 (± 6.0) years.

A total of 825 BC cases were reported in the 25-year follow-up period with the mean age at diagnosis being 70.2 (± 7.6) years. The mean follow-up of women with BC was 13.3 (± 7.2) years. In Overall, 288 (34.9%) women with BC died during the study period.

Table 1 shows the baseline characteristics of the women with BC and the entire cohort. Women with BC reported a lower work burden and more positive FH of BC than those in the reference group. Women with BC had fewer parities and used MHT for longer durations. Women with BC reported using slightly more alcohol than those without BC. Although there were more current smokers in the BC group, the pack-years smoked were lower. It had no statistical difference. (Table 1).

**Table 1:** Baseline characteristics of the study population

Variable	Breast cancer	No breast cancer	*P* value*
	n = 825	n = 11,117	
Age at baseline 1994 (years)	56.9 ± 2.8	57.3 ± 2.9	< 0.001
Age at menopause (years)	50.7 ± 4.0	49.8 ± 4.3	< 0.001
BMI (kg/m^2^)	27.5 ± 4.5	27.3 ± 4.2	0.257
Weight (kg)	72.0 ± 12.9	70.5 ± 12.2	0.001
Height (cm)	162.3 ± 5.2	161.1 ± 5.3	< 0.001
Pregnancy (n)	2.6 ± 1.8	2.9 ± 1.9	< 0.001
No pregnancies (%)	9.2%	8.4%	0.446
Parity (n)	2.4 ± 1.3	2.7 ± 1.4	< 0.001
BC family history (%)	5.6%	2.6%	< 0.001
Smoking (never) (%)	77.3%	81.1%	0.008
Current smokers (%)	15.3%	14.8%	0.759
Pack years (years)	8.3 ± 8.4	9.9 ± 10.2	0.039
work burden—low (%)	31.5%	24.9%	< 0.001
work burden—high (%)	68.3%	63.2%	< 0.001
Work career (years)	25.5 ± 11.0	24.6 ± 11.5	0.047
Alcohol consumption (g/week)	18.4 ± 30.9	15.8 ± 32.4	0.042
MHT use (yrs)	2.4 ± 3.9	1.9 ± 3.5	< 0.001

*MHT* menopausal hormone therapy

*Differences (one-way ANOVA and chi-square test)

AC was greater in the low work burden group than in the high burden group in both the reference and BC groups, although the difference was not statistically significant (Appendix 1).

Overall, 23.6% reported no occupational education in the BC group compared to 30.2% of women without BC. Women with BC reported higher educational levels than the non-BC group (Appendix 2).

### Cox regression analyses

#### Univariate

On univariate Cox regression analysis, low work burden was associated with a 1.3-fold higher risk for BC (95% confidence interval [CI] 1.2–1.6) than high work burden (Table 2).

**Table 2:** Breast cancer risk factors at baseline

Variable	Univariable^a^		Variable	Multivariable^b^	
	Cox regression			Cox regression	
	HR (95% CI)	*P* value		HR (95% CI)	*P* value
Self-rated work burden					
High (n = 8108)	1.0		High (n = 8005)	1.0	
Low (n = 3023)	1.3 (1.2–1.6)	< 0.001	Low (n = 2998)	1.3 (1.1–1.5)	0.003
Parity					
None (n = 1247)	1.0		None (n = 1224)	1.0	
Any (n = 10,411)	0.9 (0.7–1.1)	0.385	Any (n = 10,253)	0.9 (0.7–1.1)	0.382
MHT use					
Non-users (n = 7116)	1.0		Non-users (n = 6929)	1.0	
Under 5 years (n = 2745)	0.9 (0.8–1.1)	0.333	Under 5 years (n = 2692)	0.9 (0.8–1.1)	0.430
5–10 years (n = 1407)	1.5 (1.2–1.8)	< 0.001	5–10 years (n = 1388)	1.5 (1.2–1.8)	< 0.001
Over 10 years (n = 476)	1.4 (1.0–1.9)	0.026	Over 10 years (n = 468)	1.4 (1.0–1.9)	0.060
Alcohol consumption					
Abstainers (n = 4440)	1.0		Abstainers (n = 4386)	1.0	
0–100 g/week (n = 5542)	1.3 (1.1–1.5)	< 0.001	0–100 g/week (n = 5486)	1.2 (1.0–1.4)	0.014
Over 100 g/week (n = 203)	1.8 (1.2–2.9)	0.009	Over 100 g/week (n = 200)	1.6 (1.0–2.6)	0.038
Family history of breast cancer					
Negative (n = 10,241)	1.0		Negative (n = 10,070)	1.0	
Positive (n = 338)	2.0 (1.5–2.7)	< 0.001	Positive (n = 334)	1.9 (1.4–2.6)	< 0.001

*HR* hazard ratio, *95% CI* 95% confidence interval, *MHT* menopausal hormone therapy

^a^Univariate Cox regression analysis

^b^Multivariable Cox regression analysis adjusted with age and BMI (kg/m^2^)

The role of positive FH was associated with a 2.0-fold higher risk for BC (95% CI 1.5–2.7) compared to those with no maternal BC history.

Higher alcohol consumption at baseline was associated with BC. A strong association was within the weekly AC of 0–100 g (HR 1.3; 95% CI 1.1–1.5). In addition, the BC risk was elevated 1.8 -fold (95% CI 1.2–2.9) if the AC was over 100 g per week compared to abstainers.

Prolonged use of MHT was associated with an elevated risk of BC. Especially in MHT users for 5–10 years and over 10 years, with an HR of 1.5 (95% CI 1.2–1.8) and 1.4 (95% CI 1.0–2.0) compared to nonusers, respectively. MHT usage for < 5 years did not play a significant role in BC risk.

Parity (none vs. any) did not play a significant role in the univariate analysis of BC risk.

### Multivariable Cox regression

Self-rated work burden, MHT usage, alcohol consumption, FH of BC, parity, age, and BMI at baseline were used in the multivariable Cox regression analysis model. Low work burden was also associated with increased BC risk with HR 1.3 (95% CI 1.1–1.5) in the multivariable analysis. The strongest association with BC risk was positive FH (HR 1.9 95% CI 1.4–2.6) and MHT use of 5–10 years (HR 1.5-fold 95% CI 1.2–1.8). Moderate AC under 100 g/week was associated with a 1.2-fold (95% CI 1.0–1.4) and heavy AC over 100 g/week with a 1.6-fold (95% CI 1.0–2.6) higher risk of BC. (Table 2).

## Discussion

We observed that a low work burden was associated with a higher risk of BC in a large 25-year prospective, population-based study. In addition, positive FH and prolonged MHT seemed to be the most remarkable risk factors for BC among our cohort of 11,942 Finnish postmenopausal women.

Retirement age is increasing worldwide [12]. In particular, in the 53–62 years age group, working careers are longer than in the past [13]. Studies on the effects of self-rated work burden on BC risk are limited. Our study is one of the few long-term studies exploring the relationship between work burden and BC risk over a 25-year follow-up. In addition, we adjusted for several potentially confounding factors.

A sedentary lifestyle is known to be associated with several types of cancer, including BC [4]. Obesity is associated with increased BC and mortality [3, 4]. In contrast, physical activity (PA) contributes to risk reduction in several cancers, including postmenopausal BC [3–5]. Sedentary behavior is defined as any waking behavior characterized by an energy expenditure of less than 1.5 metabolic equivalents (METs), while in a sitting, reclining, or lying posture [16]. Occupational sedentary behavior comprises approximately 60% of daily sedentary behavior [17]. The role of sedentary work in BC has been conflicting, either increasing [8] or having no association with the risk [9]. According to the World Health Organization (WHO), in 2016, globally 28% of adults were not active enough [18]. It seems that the incidence of BC is lower in occupations involving higher levels of physical activity [19]. PA contributes a greater BC risk reduction among postmenopausal women who are normal weight or overweight compared to obese women (BMI ≥ 30 kg/m^2^) [5]. PA is thought to have anti-inflammatory effects while helping to maintain normal weight and reduce the levels of sex hormones such as estradiol [4, 20]. Controversially, in our study, BMI was not associated with breast cancer, which might be due to the similarity of the mean BMI between the two groups.

Night shift work has been shown to increase the BC risk in women [10, 21]. For postmenopausal women, the BC risk was not evident, suggesting that night shift work increases the risk of BC in premenopausal women [22]. Among long-term night shift workers, a greater risk was not observed among those with over 5 years of work experience in the study by Sweeney et al. [23] or over 10 years of work experience in the meta-analyses of Manouchehri et al. [24]. In a Finnish cohort study by Härmä et al., the BC risk was increased among women aged 50 years or older, in shift workers with and without night shifts, after 10 years or more [11]. In our study population, the working careers were approximately 25 years. However, our data did not consider shift work or the possible night shifts of women, which can be considered a weakness of this study. These factors may have changed during the long study period. Work exposure was recorded only at the baseline. However, the women in the present study were near retirement age, so expectedly quite a few of them had changed work burdens.

Dong et al. suggested that a higher educational level might be associated with an increased BC risk [25]. Recently, it has been shown that higher socioeconomic status is associated with a higher incidence of BC [26]. In our study, higher educational levels and occupations with higher socioeconomic status were represented more frequently in the BC group, which points in a similar direction.

BC risk is associated with several reproductive factors such as early age at menarche, late age at first pregnancy, late menopause, and low parity [6]. Among other lifestyle factors, AC and MHT usage are associated with elevated BC risk [3, 27]. It has been shown that MHT usage for 5 years increases BC risk, with the risk being twice as high with an MHT of 10 years or more [28]. Our results point in a similar direction; however, the effect is no longer significant with the use of MHT for more than 10 years with other confounding factors. Our results did not consider MHT regimens or the route of administration and were based on self-reported use, which can introduce some bias into the results. However, a previous validation study using the same cohort showed that postal inquiry is a reliable method for recording long-term MHT use in Finnish postmenopausal women [29].

A positive association between AC and the risk of BC has been observed for decades [30]. Park et al. showed that light to moderate AC increases the risk of BC in several ethnic groups [31]. A positive dose-risk relationship was observed between AC and BC [32]. The mechanisms linking alcohol intake and a higher risk of BC are thought to involve alcohol-induced elevated estrogen levels, oxidative stress, or epigenetic changes with an altered cell cycle [32]. Our findings are consistent with those of previous studies. We did not observe a significant relationship between BC and heavy AC, which might be due to the small sample size (n = 203), as well as the possible unwillingness of heavy alcohol users to report their actual consumption.

Positive FH was associated with the highest risk of BC risk among the factors. An elevated risk is associated with a positive FH, especially in younger women [33]. This risk appears to be elevated in postmenopausal women as well [34]. Our findings outlined the impact of positive FH on BC risk.

Although occupations were widely represented in our study population, they all originated from the same geographical district and same age range in Finland. This makes the study population homogenous but may limit the generalization of the results to the entire population.

In conclusion, low work burden is associated with postmenopausal BC after adjusting for other confounding factors such as MHT, maternal history of breast cancer, and alcohol use. Further long-term studies are needed to investigate the influence of work burden on the risk of postmenopausal BC.

## Data Availability

The datasets generated / or analyzed during the current study are available from the corrsponding author on a reasonable request.

## Appendix 1

**Table:** The association of breast cancer with amount of alcohol use and self-rated work burden

	Self-rated work burden			
	Low HR (95% CI)^a^	*P* value	High HR (95% CI)	*P* value
Alcohol consumption (g/week)	n = 260		n = 521	
Abstainers (n = 4440)	1.0		1.0	
Moderate—0 to 100 (n = 5542)	1.3 (0.9–1.7)	0.114	1.3 (1.0–1.5)	0.021
Heavy—over 100 (n = 203)	1.4 (0.7–2.8)	0.370	2.1 (1.2–3.9)	0.015

^a^HR, hazard ratio with 95% confidence interval; Cox regression analysis

## Appendix 2

**Table:** Socioeconomic characteristics of the study population at baseline

	Breast Cancer	No breast cancer	*P* value*
	n = 825	n = 11,117	
Retired (full-time)	3.9%	4.9%	0.003
Working (full time)	47.7%	41.6%	0.003
Unemployed	13.3%	12.2%	0.003
Unable to work—not retired	3.9%	2.9%	0.003
Disability retirement	18.0%	22.0%	0.003
Education			
Elementary school	60.1%	70.6%	< 0.001
Comprehensive school	25.2%	17.5%	< 0.001
High school	12.3%	9.0%	< 0.001
Occupational education			
No occupational education	23.6%	30.2%	< 0.001
Career college	14.8%	14.3%	< 0.001
University/college	7.1%	6.3%	< 0.001
Occupation			
Doctor/health care	11.1%	7.4%	< 0.001
Teaching	6.5%	5.5%	< 0.001
Farm worker	14.2%	16.8%	< 0.001
Household/domestic work	7.2%	9.0%	< 0.001
Cleaning/maintenance	8.8%	10.9%	< 0.001
Office work	9.3%	6.6%	< 0.001

*Differences between groups (chi-square test)

## Abbreviations

BC: Breast cancer
MHT: Menopausal hormone therapy
AC: Alcohol consumption
BMI: Body mass index
FH: Family history
HR: Hazard ratio

## References

[1] World Health Organization—WHO. International Agency for Research on Cancer 2020. https://gco.iarc.fr. Accessed 15 Jan 2021

[2] Global Burden of Disease 2019 Cancer Collaboration (2022) Cancer incidence, mortality, years of life lost, years lived with disability, and disability-adjusted life years for 29 cancer groups from 2010 to 2019: a systematic analysis for the global burden of disease study 2019. JAMA Oncol 8(3):420–444. 10.1001/jamaoncol.2021.6987 10.1001/jamaoncol.2021.6987 PMC8719276 34967848

[3] Ellingjord-Dale M, Vos L, Vik Hjerkind K, Hjartåker A, Russnes HG, Tretli S, Hofvind S, Dos-Santos-Silva I, Ursin G (2018) Number of risky lifestyle behaviors and breast cancer risk. JNCI Cancer Spectrum 2(3):pky030. 10.1093/jncics/pky30 31360858 10.1093/jncics/pky030 PMC6649737

[4] Kerr J, Anderson C, Lippman S (2017) Physical activity, sedentary behaviour, diet and cancer: an update and emerging new evidence. Lancet Oncol 18:e457–e471. 10.1016/S1470-2045(17)30411-4 28759385 10.1016/S1470-2045(17)30411-4 PMC10441558

[5] Neil-Sztramko SE, Boyle T, Milosevic E, Nugent SF, Gotay CC, Campbell KL (2017) Does obesity modify the relationship between physical activity and breast cancer risk? Breast Cancer Res Treat 166:367–281. 10.1007/s10549-017-4449-4 28803384 10.1007/s10549-017-4449-4

[6] McCredie M, Paul C, Skegg DC, Williams S (1998) Reproductive factors and breast cancer in New Zealand. Int J Cancer 76(2):182–8. 10.1002/(sici)1097-0215(19980413)76:2%3c182::aid-ijc3%3e3.0.co;2-t 9537578 10.1002/(sici)1097-0215(19980413)76:2<182::aid-ijc3>3.0.co;2-t

[7] Wylenzek F, Bühling KJ, Laakmann E (2024) A systematic review on the impact of nutrition and possible supplementation on the deficiency of vitamin complexes, iron, omega-3-fatty acids, and lycopene in relation to increased morbidity in women after menopause. Arch Gynecol Obstet 310(4):2235–2245. 10.1007/s00404-024-07555-6 38935105 10.1007/s00404-024-07555-6 PMC11393286

[8] Lee J, Lee J, Lee DW, Kim HR, Kang MY (2021) Sedentary work and breast cancer risk: a systematic review and meta-analysis. J Occup Health 63(1):e12239. 10.1002/1348-9585.12239 34161650 10.1002/1348-9585.12239 PMC8221371

[9] Boyle T, Fritschi L, Kobayashi LC, Heyworth JS, Lee DG, Si S, Aronson KJ, Spinelli JJ (2016) Sedentary work and the risk of breast cancer in premenopausal and postmenopausal women: a pooled analysis of two case-control studies. Occup Environ Med 73(11):735–741. 10.1136/oemed-2015-103537 27540104 10.1136/oemed-2015-103537

[10] Megdal SP, Kroenke CH, Laden F, Pukkala E, Schernhammer ES (2005) Night work and breast cancer risk: a systematic review and meta-analysis. Eur J Cancer 41(13):2023–2032. 10.1016/j.ejca.2005.05.010 16084719 10.1016/j.ejca.2005.05.010

[11] Härmä M, Ojajärvi A, Koskinen A, Lie JA, Hansen J (2022) Shift work with and without night shifts and breast cancer risk in a cohort study from Finland. Occup Environ Med. 10.1136/oemed-2022-108347 35948413 10.1136/oemed-2022-108347 PMC9763178

[12] OECD (2019) Will future pensioners work on longer and retire less? Policy Brief on Pensions. OECD Publishing, Paris

[13] Nivalainen S. Socio-economic differences: retirement and working lives in 2006, 2011 and 2017. Finnish Centre for Pensions, Studies. https://urn.fi/URN:ISBN:978-951-691-347-9

[14] Leinonen MK, Miettinen J, Heikkinen S, Pitkäniemi J, Malila N (2017) Quality measures of the population-based Finnish Cancer Registry indicate sound data quality for solid malignant tumours. Eur J Cancer 77:31–39. 10.1016/j.ejca.2017.02.017 28350996 10.1016/j.ejca.2017.02.017

[15] Alcohol abuse. Current Care Guidelines. Working group set up by the Finnish Medical Society Duodecim and the Finnish Society of Addiction Medicine. Helsinki: The Finnish Medical Society Duodecim, 2015 (referred December 19, 2022). www.kaypahoito.fi

[16] Tremblay MS, Aubert S, Barnes JD, Saunders TJ, Carson V, Latimer-Cheung AE, Chastin SFM, Altenburg TM, Chinapaw MJM, SBRN Terminology Consensus Project Participants (2017) Sedentary behavior research network (SBRN)—terminology consensus project process and outcome. Int J Behav Nutr Phys Act 14(1):75. 10.1186/s12966-017-0525-8 28599680 10.1186/s12966-017-0525-8 PMC5466781

[17] Bailey DP (2021) Sedentary behaviour in the workplace: prevalence, health implications and interventions. Br Med Bull 137(1):42–50. 10.1093/bmb/ldaa039 33710270 10.1093/bmb/ldaa039

[18] World Health Organization (2020) Physical inactivity: a global public health problem. World Health Organization, Geneva. https://www.who.int/dietphysicalactivity/factsheet_inactivity/en/. Accessed 13 Sept 2022

[19] Pukkala E, Martinsen JI, Lynge E, Gunnarsdottir HK, Sparén P, Tryggvadottir L, Weiderpass E, Kjaerheim K (2009) Occupation and cancer—follow-up of 15 million people in five Nordic countries. Acta Oncol 48(5):646–790. 10.1080/02841860902913546 19925375 10.1080/02841860902913546

[20] Friedenreich CM, Ryder-Burbidge C, McNeil J (2021) Physical activity, obesity and sedentary behavior in cancer etiology: epidemiologic evidence and biologic mechanisms. Mol Oncol 15(3):790–800. 10.1002/1878-0261.12772 32741068 10.1002/1878-0261.12772 PMC7931121

[21] Gehlert S, Clanton M, On Behalf Of The Shift Work And Breast Cancer Strategic Advisory Group (2020) Shift work and breast cancer. Int J Environ Res Public Health 17(24):9544. 10.3390/ijerph17249544 33419321 10.3390/ijerph17249544 PMC7767214

[22] Cordina-Duverger E, Menegaux F, Popa A, Rabstein S, Harth V, Pesch B, Brüning T, Fritschi L, Glass DC, Heyworth JS, Erren TC, Castaño-Vinyals G, Papantoniou K, Espinosa A, Kogevinas M, Grundy A, Spinelli JJ, Aronson KJ, Guénel P (2018) Night shift work and breast cancer: a pooled analysis of population-based case-control studies with complete work history. Eur J Epidemiol 33(4):369–379. 10.1007/s10654-018-0368-x 29464445 10.1007/s10654-018-0368-x

[23] Sweeney MR, Sandler DP, Niehoff NM, White AJ (2020) Shift work and working at night in relation to breast cancer incidence. Cancer Epidemiol Biomark Prev 29(3):687–689. 10.1158/1055-9965.EPI-19-1314 10.1158/1055-9965.EPI-19-1314 PMC7060110 31915142

[24] Manouchehri E, Taghipour A, Ghavami V, Ebadi A, Homaei F, Latifnejad RR (2021) Night-shift work duration and breast cancer risk: an updated systematic review and meta-analysis. BMC Womens Health 21(1):89. 10.1186/s12905-021-01233-4 33653334 10.1186/s12905-021-01233-4 PMC7927396

[25] Dong J, Qin L (2020) Education level and breast cancer incidence: a meta-analysis of cohort studies. Menopause 27(1):113–118. 10.1097/GME.0000000000001425 31479033 10.1097/GME.0000000000001425

[26] Tweel M, Johnston GM, Hajizadeh M (2023) Trends in socioeconomic inequalities in breast cancer incidence among women in Canada. Cancer Control 30:10732748231197580. 10.1177/10732748231197580 37608582 10.1177/10732748231197580 PMC10467209

[27] Temkin SM, Mallen A, Bellavance E, Rubinsak L, Wenham RM (2019) The role of menopausal hormone therapy in women with or at risk of ovarian and breast cancers: misconceptions and current directions. Cancer 125(4):499–514. 10.1002/cncr.31911 30570740 10.1002/cncr.31911

[28] Collaborative Group on Hormonal Factors in Breast Cancer (2019) Type and timing of menopausal hormone therapy and breast cancer risk: individual participant meta-analysis of the worldwide epidemiological evidence. Lancet 394(10204):1159–1168. 10.1016/S0140-6736(19)31709-X 31474332 10.1016/S0140-6736(19)31709-X PMC6891893

[29] Sandini L, Pentti K, Tuppurainen M, Kröger H, Honkanen R (2008) Agreement of self-reported estrogen use with prescription data: an analysis of women from the Kuopio Osteoporosis Risk Factor and Prevention Study. Menopause 15(2):282–9. 10.1097/gme.0b013e3181334b6c 17998884 10.1097/gme.0b013e3181334b6c

[30] Rosenberg L, Slone D, Shapiro S, Kaufman DW, Helmrich SP, Miettinen OS, Stolley PD, Levy M, Rosenshein NB, Schottenfeld D, Engle RL Jr (1982) Breast cancer and alcoholic-beverage consumption. Lancet 1(8266):267–270. 10.1016/s0140-6736(82)90987-4 6120284 10.1016/s0140-6736(82)90987-4

[31] Park SY, Kolonel LN, Lim U, White KK, Henderson BE, Wilkens LR (2014) Alcohol consumption and breast cancer risk among women from five ethnic groups with light to moderate intakes: the Multiethnic Cohort Study. Int J Cancer 134(6):1504–1510. 10.1002/ijc.28476 24037751 10.1002/ijc.28476 PMC4102309

[32] Seitz HK, Pelucchi C, Bagnardi V, La Vecchia C (2012) Epidemiology and pathophysiology of alcohol and breast cancer: update 2012. Alcohol 47(3):204–12. 10.1093/alcalc/ags011 10.1093/alcalc/ags011 22459019

[33] Shiyanbola OO, Arao RF, Miglioretti DL, Sprague BL, Hampton JM, Stout NK, Kerlikowske K, Braithwaite D, Buist DSM, Egan KM, Newcomb PA, Trentham-Dietz A (2017) Emerging trends in family history of breast cancer and associated risk. Cancer Epidemiol Biomark Prev 26(12):1753–1760. 10.1158/1055-9965.EPI-17-0531 10.1158/1055-9965.EPI-17-0531 PMC5712247 28986348

[34] Braithwaite D, Miglioretti DL, Zhu W, Demb J, Trentham-Dietz A, Sprague B, Tice JA, Onega T, Henderson LM, Buist DSM, Ziv E, Walter LC, Kerlikowske K, Breast Cancer Surveillance Consortium (2018) Family history and breast cancer risk among older women in the Breast Cancer Surveillance Consortium Cohort. JAMA Intern Med 178(4):494–501. 10.1001/jamainternmed.2017.8642 29435563 10.1001/jamainternmed.2017.8642 PMC5876845

